# Lithopaedion

**Published:** 1914-05

**Authors:** 


**Lithopaedion.** The Lancet, Dec. 27, 1913, records a Toronto case, retained 42 years, during four succeeding normal pregnancies and finally removed by laparotomy.

